# The hallucal interphalangeal ossicle: anatomy and basis for ultrasound-guided surgical shaving

**DOI:** 10.1038/s41598-022-08805-w

**Published:** 2022-03-21

**Authors:** Simone Moroni, Javier Márquez, Alejandro Fernández-Gibello, Gabriel Camunas Nieves, Ruben Montes, Teresa Vázquez, José Ramon Sanudo, Bernhard Moriggl, Carla Stecco, R. Shane Tubbs, Marko Konschake

**Affiliations:** 1 Catholic University Saint Vincent Martir, Faculty of Medicine, Valencia, Spain; 2 Central University of Cataluna (VIC), Faculty of Medicine, VIC, Spain; 3 Department of Podiatry, Faculty of Health Sciences, University of La Salle, Clinic Vitruvio, Madrid, Spain; 4 Anatomy and Embryology Department, School of Medicine, Complutense University of Madrid, Madrid, Spain; 5 Department of Anatomy, Histology and Embryology, Institute of Clinical and Functional Anatomy, Medical University of Innsbruck (MUI), Müllerstr. 59, 6020 Innsbruck, Austria; 6 Department of Neurosciences, Institute of Human Anatomy, University of Padova, Padua, Italy; 7 Department of Neurosurgery, Tulane Center for Clinical Neurosciences, Tulane University School of Medicine, New Orleans, LA USA; 8 Department of Neurology, Tulane Center for Clinical Neurosciences, Tulane University School of Medicine, New Orleans, LA USA; 9 Department of Anatomical Sciences, St. George’s University, West Indies, St. George’s, Grenada; 10 Department of Structural and Cellular Biology, Tulane University School of Medicine, New Orleans, LA USA; 11 Department of Neurosurgery and Ochsner Neuroscience Institute, Ochsner Health System, New Orleans, LA USA; 12 Department of Surgery, Tulane University School of Medicine, New Orleans, LA USA; 13 University of Queensland, Brisbane, Australia

**Keywords:** Translational research, Bone, Preclinical research

## Abstract

Painful lesions on the plantar aspect of the first interphalangeal joint (IPJ) of the hallux can be attributed to structures called ossicles, nodules, or sesamoids. The aims of the present study were first to verify that ultrasonography (US) is a high-sensitivity tool for diagnosing an interphalangeal ossicle (IO), and second to prove that US-guided-shaving surgery (“milling”) is a safe and feasible technique for remodeling the IO. The study is divided into three parts. In the first part, the prevalence of IOs was estimated in 12 cadaver feet using US, anatomical dissection, and fluoroscopy. In the second, a detailed US and morphological description of the IO was obtained. In the third, six cadaver feet were subjected to surgical milling. IO prevalence was 41.6% in gross anatomy, 41.6% in US examination and just 16.6% in fluoroscopy. The ossicles had a mean length of 4 mm (± 2 mm) and a width of 7 mm (± 2 mm). The ossicles could be completely shaved in all specimens without injuring important anatomical structures. Our results indicate that US is a more precise tool for diagnosing an IO than X-ray. Moreover, our US-guided mini-invasive surgical technique appears feasible and safe.

## Introduction

Painful lesions on the plantar aspect of the first interphalangeal joint (IPJ) of the hallux can be attributable to anatomical structures called ossicles, nodules, or (inconstant) sesamoid bones^1–7^. Our literature search showed that such structures have been named differently owing to their location, morphology, and histology^6–8^. Previously, these ossicles were considered to be sesamoids or sesamoid bones because of their origin, lying encapsulated in the fibers of the flexor hallucis longus tendon (FHL) or within the structure of a tendon called “flexor hallucis capsularis interphalangeus” (FHCI)^7^. Later studies suggested that these ossicles are included in the IPJ plantar plate capsule^1^. Therefore, the correct current terminology and definition should be “interphalangeal ossicle” (IO)^1^. The reported prevalence of the IO is three times higher in dissection studies (up to 71.6%) than in-plane X-rays studies (up to 22.8%)^9^. Yammine concluded that the IO cannot be seen in radiological projections or, more likely, that these IO “bones” are not sufficiently ossified, being mostly composed of fibrocartilage and often obscured by the opacity of larger bones^9^. Imaging procedures such as magnetic resonance imaging, bone scintigraphy, or CT should be considered if IO is hypothesized as the etiology of chronic hallucal plantar pain^10^. US has been used as a first diagnostic tool in many musculoskeletal examinations: in 2005, Brigido et al. showed that accessory sesamoids such as IO, both ossified and not fully ossified, have a typical appearance on US^11^. To the best of our knowledge, no research has been reported comparing US and X-rays for reliability of diagnosis of pathologies due to IOs, and neither the feasibility nor the safety of US-guided IO surgical shaving has been assessed. Therefore, the aims of this preclinical study were to ascertain that US-guided shaving of the IO is an effective and safe procedure in interphalangeal ossicleplasty (or its exeresis) and to determine whether the reported prevalence of the IO is higher using US than X-rays, compared with dissection.

## Material and methods

This study was carried out on 12 feet of fresh frozen, caucasian body-donors to science. The feet were donated to the anatomical department of Complutense University of Madrid (Body Donation Center). The individuals had given their written informed consent before death for use for scientific and educational purposes. Under national law, scientific institutions (generally medical university institutes, departments, or divisions) are entitled to receive the body after death primarily through a specific legacy, which is a special form of last will and testament. Legacies are not accepted without the donor having recorded his legacy and given the appropriate information on which to make a decision based on written informed consent (ethics policy). Therefore, approval by the ethics committee was not necessary^12,13^.

The exclusion criteria for this study were: previous history of forefoot surgery, and space occupying lesions in the hallux. The included cadavers were between 18 and 90 years of age.

The study was divided into three parts. In the first part, the 12 feet were examined by US and fluoroscopy to establish the prevalence of IOs. In the second, a detailed US description of the examined area was obtained and the IOs were measured. To ascertain the real prevalence and to measure the gross anatomy of the IOs, the feet were anatomically dissected. The third part consisted in performing the surgical IO shaving technique (“milling”) on six feet under US guidance by a podiatric surgeon with more than 7 years’ experience of mini-invasive US-guided surgical procedures. After the technique was performed, a clinical anatomist with more than 15 years’ experience dissected the feet to verify the procedure and establish that no important adjacent anatomical structures were injured.

### First part

#### Instruments

For the first part of the study a US machine (Sonoscape P-50, Italy) with a high frequency linear probe (12–18 MHz) and a fluoroscopy machine (Siemens, Spain) were used.

#### Method

All feet were examined by US in long and short axis with respect to the IPJ, which allowed the IO to be verified and measured. Subsequently, all 12 feet were examined by fluoroscopy in a lateral specific IPJ projection.

### Second part

#### Instruments

For the second part of our study, a No.3 scalpel handle with No. 10 and 15 blades, dissection forceps with teeth, and dissection scissors were required.

#### Method

The donated bodies were dissected to make detailed anatomical evaluations of the joint; photographs were taken to objectify the anatomical relationships of the IO. After the IO was dissected, its gross anatomical dimensions were compared to those obtained from US examination.

### Third part

#### Instruments

To perform the surgical technique, a US machine (Sonoscape P-50, Italy) with a high frequency linear probe (12–18 MHz), an 18 g 40 mm needle with a 20 cc syringe and saline solution, a Beaver scalpel with a 64 mis blade, a blunt freer-type dissector, a minimally invasive surgery (MIS) micromotor with a 20:1 reductor, a Shannon mini burr and a mini Polokoff type miller were used.

#### “Step-by step” approach

The IO was located under US guidance on its long and short axes, its dimensions were measured, and a dorsal approach to the medial plantar nerve was performed.

The needle was inserted between the non-articular plantar side of the IO and the dorsal surface of the FHL. Hydrodissection was conducted in a fan-shape fashion, with an18g 40 mm needle adapted to a 20 cc syringe filled with saline. The incision (1.5 mm) was made with a 64 mis scalpel.

**Figure 1:**
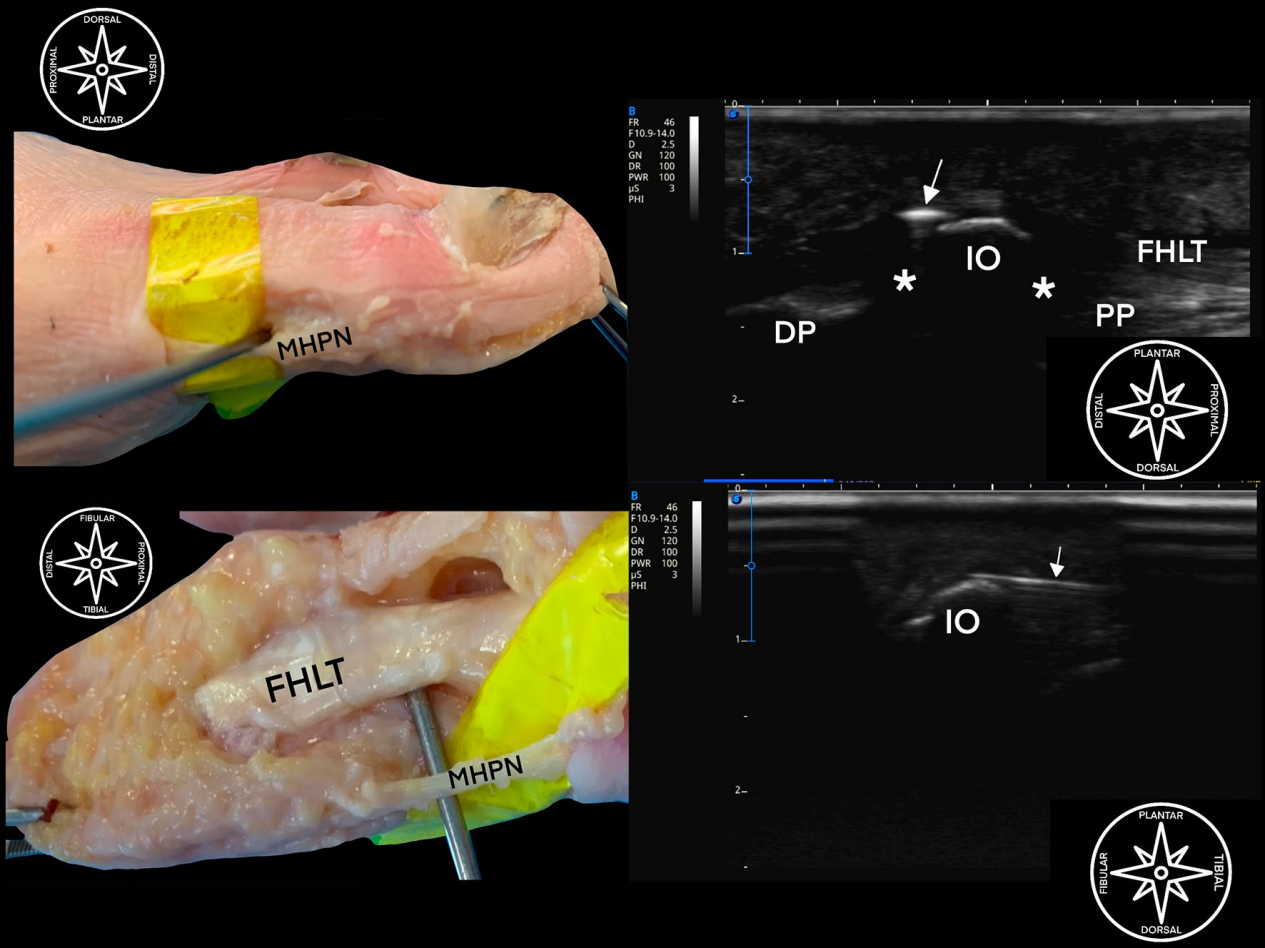
Ultrasound guided shaving of the interphalangeal joint ossicle of a left foot. (**A**) Dissection representation (tibial aspect) showing minimally invasive burr just dorsal to MHPN (medial plantar nerve). (**B**) Ultrasound guided shaving; *PP* proximal phalanx, *DP* distal phalanx, *IO* interphalangeal ossicle, *asterisk* * plantar plate, FHLT flexor hallucis longus tendon, white arrow showing burr (out of plane) between FHL and IO. (**C**) Dissection representation (plantar aspect) showing minimally invasive burr just dorsal to MHPN (medial plantar nerve); FHLT flexor hallucis longus tendon. (**D**) Ultrasound guided shaving; *IO* interphalangeal ossicle, white arrow showing burr (in-plane) between FHLT and IO.

A blunt dissection was done with the freer elevator at the non-articular aspect of the plantar IO in a fan-shape fashion. The Shannon mini burr was then used, and the IO was shaved in the axial plane (Fig. 1).

### Ethical approval

According to the ethical statements we want to clarify that an ethics review board approval was not necessary (and available) by laws. All processes in getting these cadavers (after obtaining informed consent) are either described in the manuscript or in detail in the references^14,15^: the body-donors to science were donated to the anatomical department of the Complutense University Madrid, Spain. The individuals had given their written informed consent before death for use for scientific and educational purposes. Under national law, scientific institutions (generally medical university institutes, departments, or divisions) are entitled to receive the body after death primarily through a specific legacy, which is a special form of last will and testament. Legacies are not accepted without the donor having recorded his legacy and given the appropriate information on which to make a decision based on written informed consent (ethics policy). Therefore, approval by the ethics committee was not necessary^14,15^. We confirm that all methods were carried out in accordance with relevant guidelines and regulations**.**

## Results

### First part

The first part compared US, fluoroscopy, and gross anatomy. The sexes of the specimens could not be considered since the male/female ratio was unbalanced.

**Figure 2:**
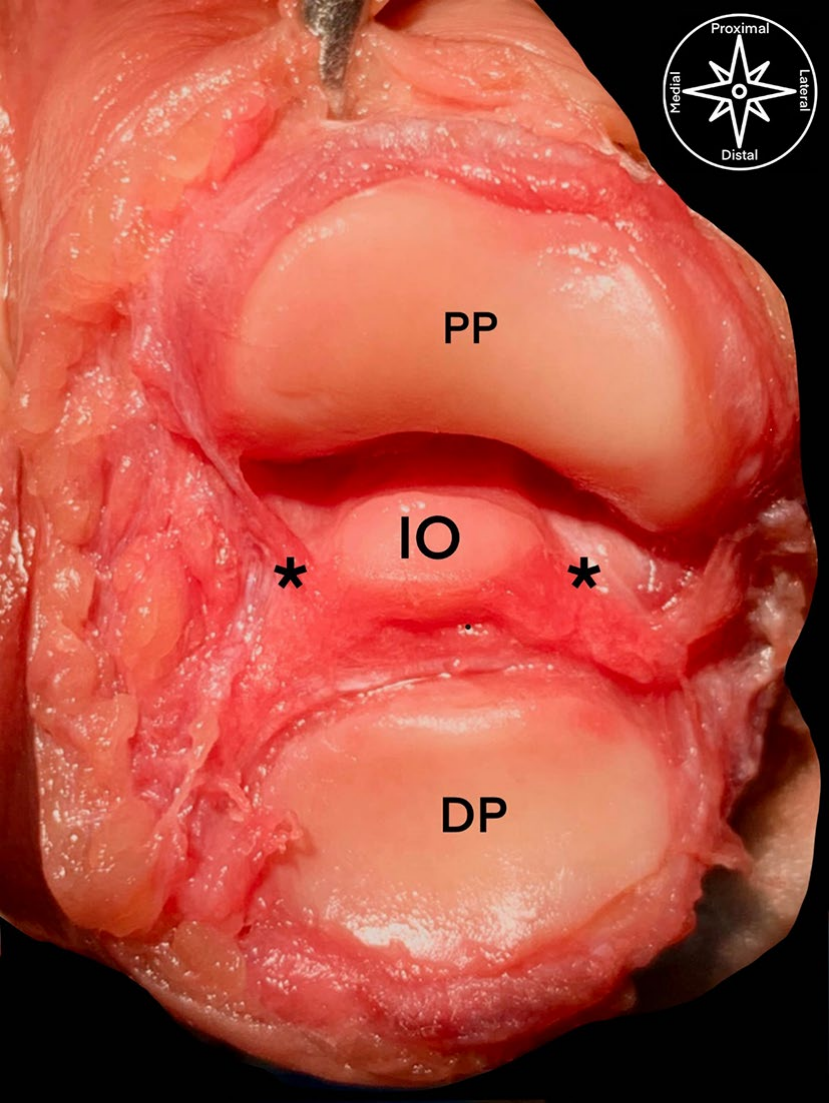
Anatomical dissection of the interphalangeal joint of a right foot from a dorsal aspect: *PP* proximal phalanx condyle, *DP* distal phalanx base, *asterisk* * plantar plate, *IO* interphalangeal ossicle.

The prevalence of the IO in our sample was 41.6% by dissection, 41.6% by US examination and 16.6% by fluoroscopy (Table 1). Figure 2 shows the dissection of a specimen with an ossified IO.

**Table 1:**
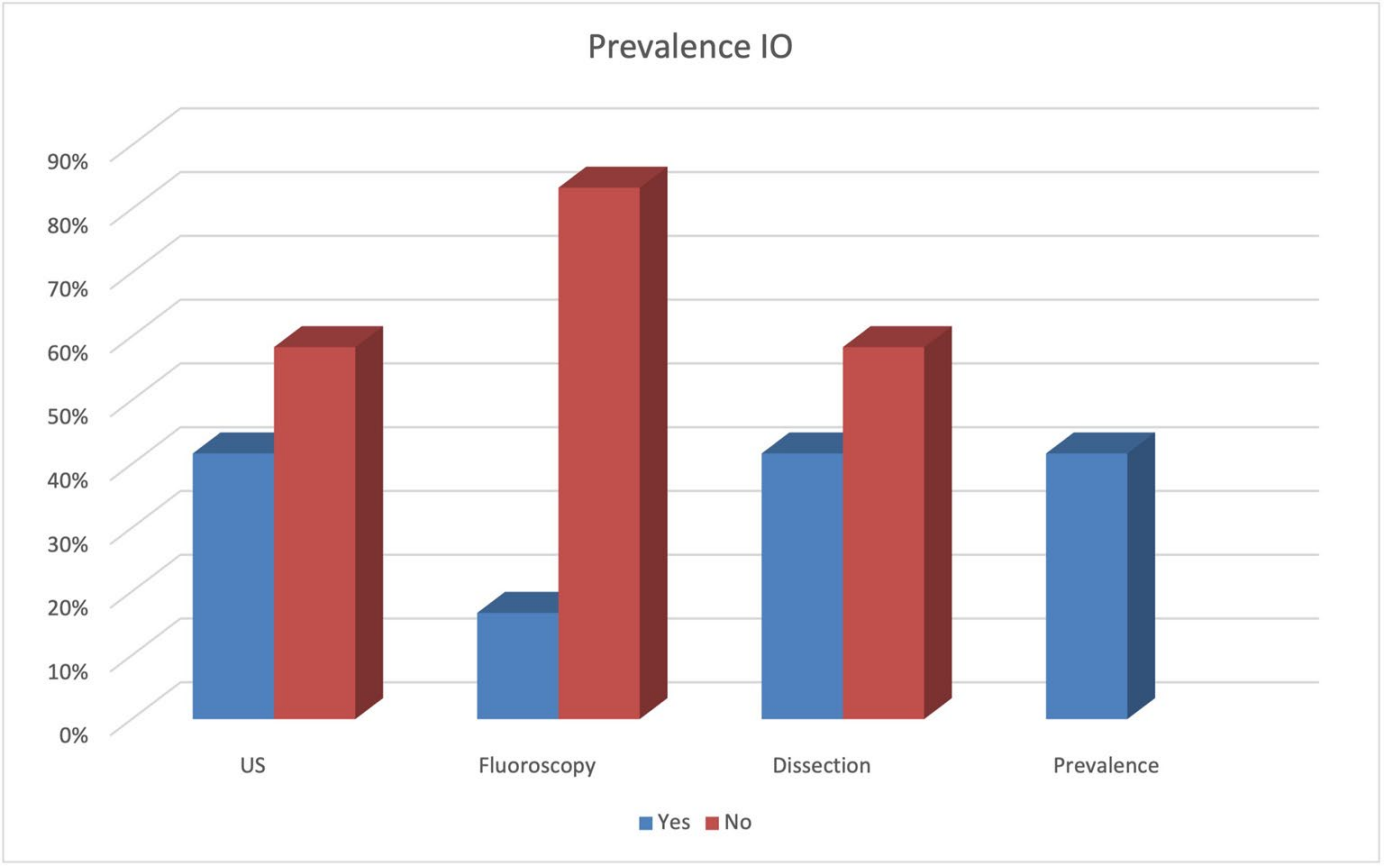
The total prevalence of hallucal interphalangeal ossicles detected including a comparison of the different methods used: ultrasonography, fluoroscopy, and dissection.

### Second part

**Figure 3:**
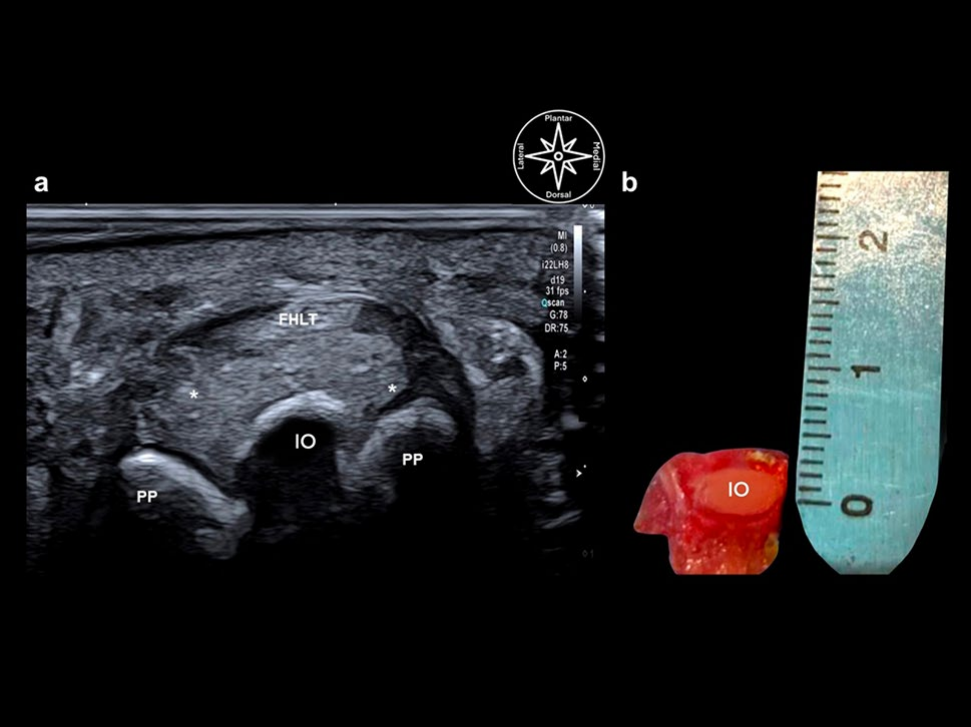
Comparison between diagnostic ultrasound (**a**) and gross anatomy of the interphalangeal ossicle in the axial view (short axis). (**b**) *PP* proximal phalanx, *IO* interphalangeal ossicle (5.8 mm), *FHLT* flexor hallucis longus tendon, asterix* plantar plate, *IO* interphalangeal ossicle (6 mm).

**Figure 4:**
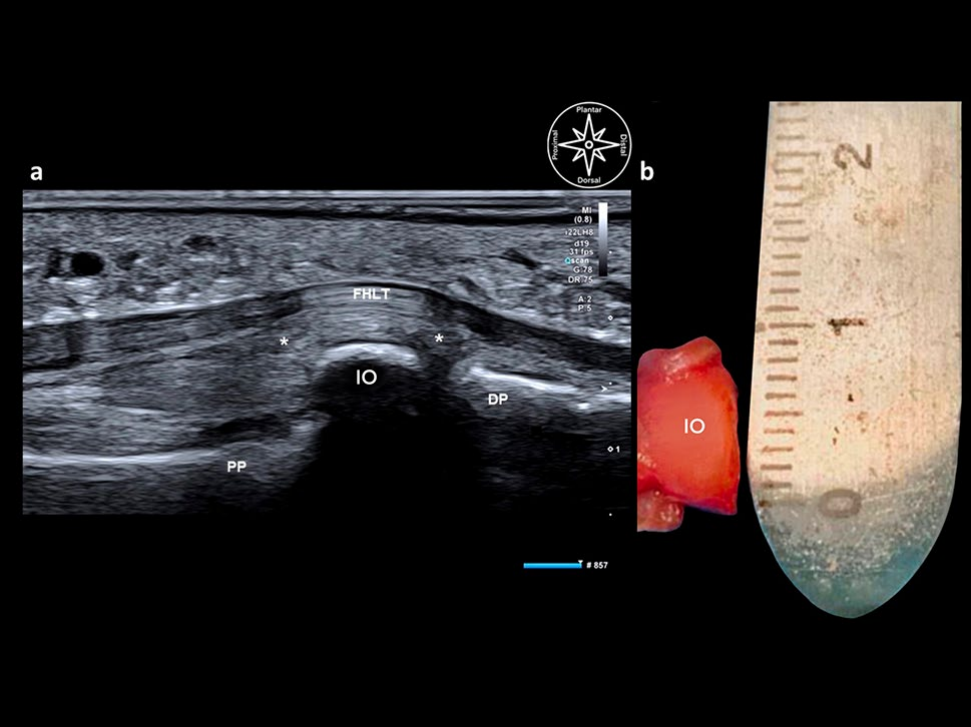
Comparison between diagnostic ultrasound (**a**) and gross anatomy of the interphalangeal ossicle in the sagittal view (long axis). (**b**): *PP* proximal phalanx, *DP* distal phalanx, *IO* interphalangeal ossicle (3.7 mm), FHLT flexor hallucis longus tendon, asterix* plantar plate, *IO* interphalangeal ossicle (6 mm).

**Figure 5:**
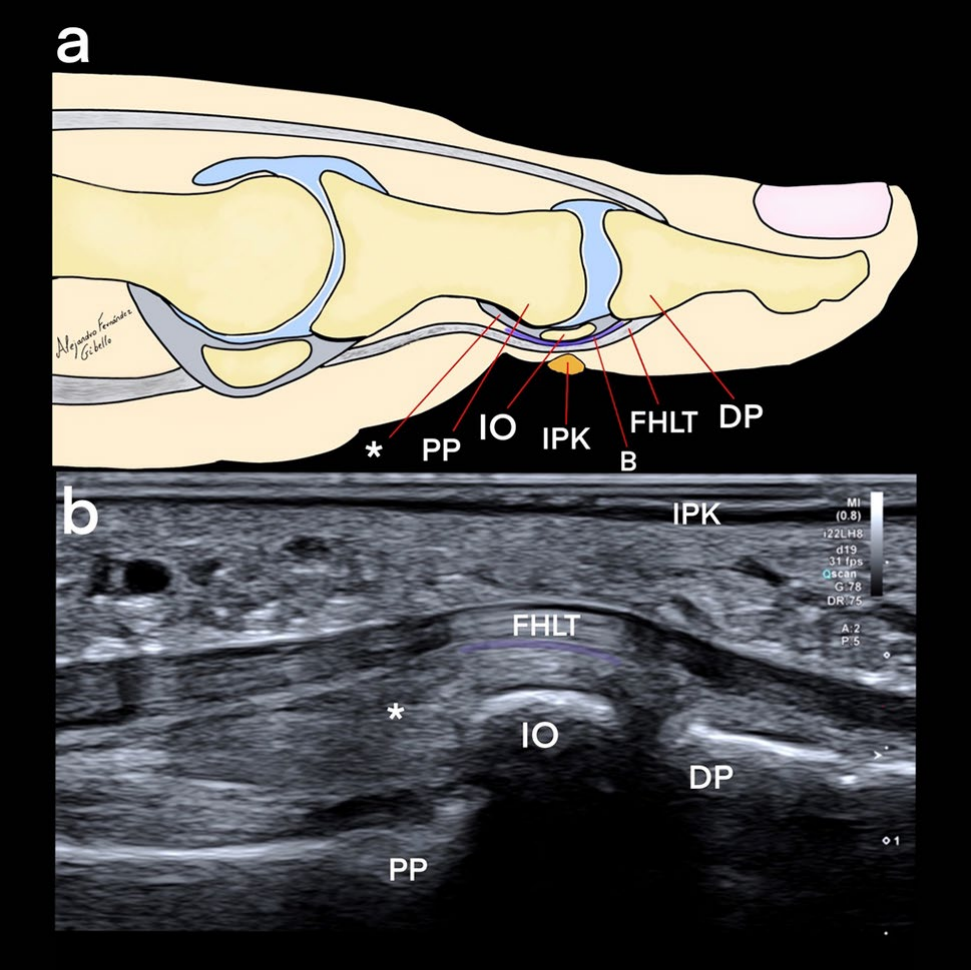
Comparison between a sketch (**a**) and an ultrasound sagittal view (**b**): asterix* plantar plate, *IO* interphalangeal ossicle, *PP* proximal phalangeal, *DP* distal phalanx, *FHLT* flexor hallucis longus tendon, *IPK* intractable plantar keratosis, *B* bursa.

Subcutaneous cellular tissue with heterogeneous echogenicity reflecting the state of aggregation of the plantar fat could be demonstrated on the plantar aspect in the long axis. Dorsally, the FHL tendon was depicted with a typical fibrillar pattern, and its fibrous sheath appeared as a thin hyperechoic band. Dorsal to the tendon, if an IO was present, a hypoechoic space corresponding to a bursa could be seen. That bursa lay between the FHL and the non-articular plantar aspect of the IO; the latter was seen as a hyperechoic convex band. This ossicle had a mean length (e.g., long axis at the IPJ) of 4 mm ± 2 mm and a width (e.g., short axis at the IPJ) of 7 mm ± 2 mm (Figs. 3, 4, 5).

The IO was embedded in the plantar plate of the IPJ, which has a moderately hyperechoic pattern typical of fibrocartilage. The plantar plate of the IPJ was inserted proximally to the condyle of the proximal phalanx and distally to the condyle of the base of the distal phalanx; both showed the same echogenicity as the non-articular plantar aspect of the IO.

**Figure 6:**
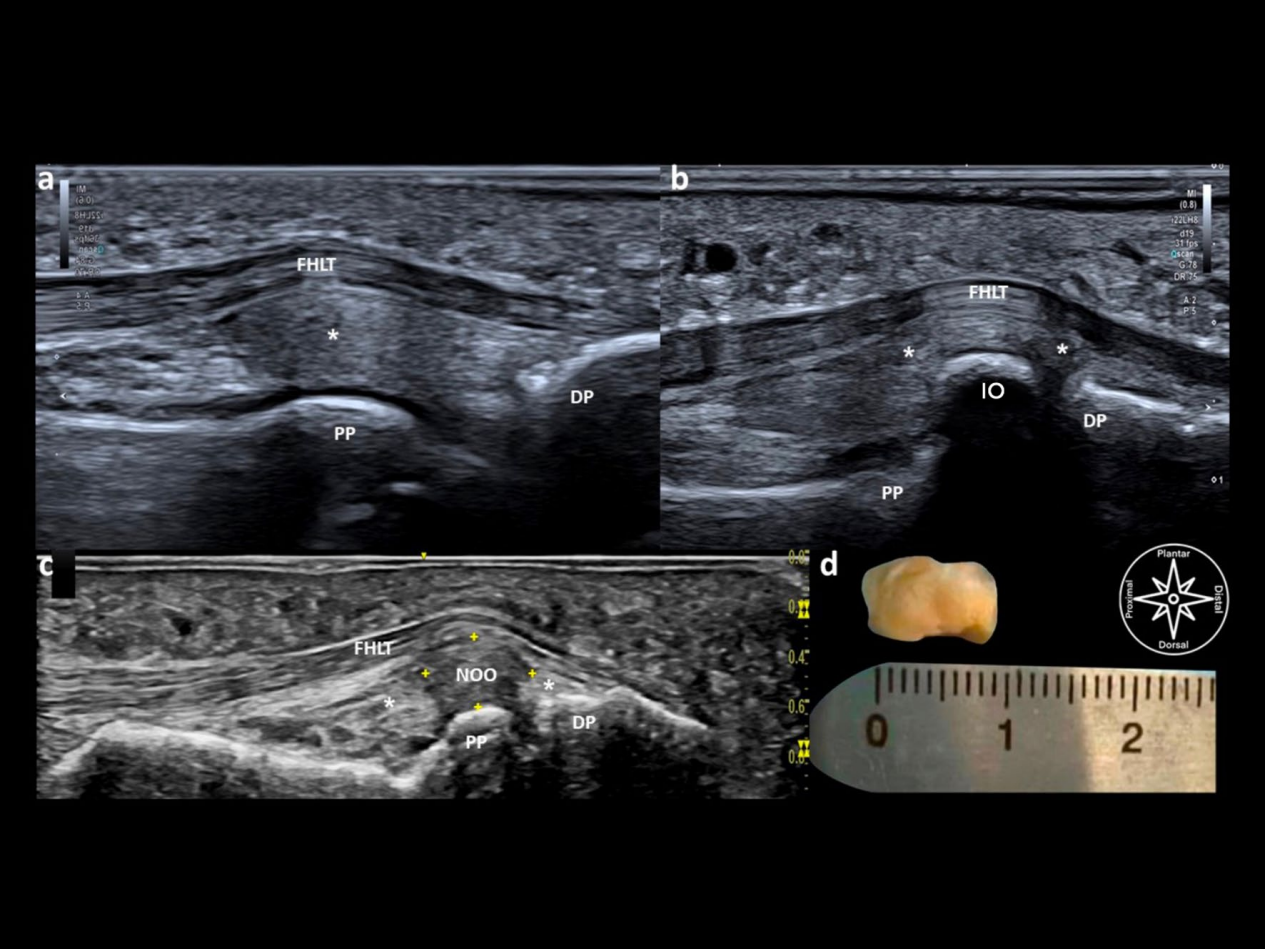
Diagnostic ultrasound findings in different plantar interphalangeal joint aspects, sagittal scanning without an ossicle (**a**), with an ossified ossicle (**b**) with a non-ossified (fibrocartilage) ossicle (**c**), comparative view of an interphalangeal non-ossified ossicle via diagnostic ultrasound (long axis measure 9 mm) and via gross anatomy body-donor (sagittal measure 9 mm) (**d**): *PP* proximal phalanx, *DP* distal phalanx, *IO* interphalangeal ossicle, *FHLT* flexor hallucis longus tendon, asterix* plantar plate, *NOO* non-ossified ossicle.

Cases of non-ossified IO (a nodular, well-defined fibrocartilage-like structure) depicted by US were always embedded in the IPJ plantar plate. When no IO could be detected, the dorsal aspect of the FHL was partially inserted to the plantar side of the plantar plate (Fig. 6).

The first and second part have been evaluated by the experienced foot and ankle surgeons together with clinical anatomists.

### Third part

After the six cadavers with ossified IOs had been subjected to shaving, the clinical anatomist started the dissections. In all feet, the results showed that the technique had been correctly performed and that all adjacent anatomical structures (medial plantar nerve, FHL tendon, plantar IPJ) were saved.

## Discussion

The hallucal IO is a frequent cause of painful lesions on the IPJ-complex^1–5,7,8^. This ossicle can be attached to multiple adjacent structures including tendons, the metatarsal basis, and the plantar fascia, together forming an IPJ-capsuloligamentous-sesamoid complex^8^.

In the first part of our study, we verified our hypothesis that US is a more sensitive diagnostic tool for a hallucal IO than fluoroscopy, which accords with the literature^16–18^. The prevalence revealed by US and dissection in our study was 41.6%, while fluoroscopy only detected an IO in 16.6% (39.9% accuracy in fluoroscopy while US has 100%). Other groups such as Davies and Dalal and Suwannahoy et al. showed a prevalence of up to 88% over 100 IPJs dissections^1,19^. A meta-analysis and systematic review by Yammine in 2014 showed quite similar results, reporting a prevalence of 71.6% in dissections but only 22.8% in plain X-rays^9^. Owing to the sample size, the author’s objective was not to describe the prevalence of the IO over the general population, which would have been unavoidably biased, but to establish the accuracy of diagnosis by comparing the prevalence between US, plain X-rays and dissection.

In three of our samples examined with US, the IOs seemed only partially ossified. They could not be visualized by subsequent fluoroscopy. Dissection of these three cases also revealed partial ossification. These results confirm that US is a much more sensitive tool for IO diagnosis than fluoroscopy. In cases in which the IO appeared not to be ossified, it could still be diagnosed by US owing to its typical appearance: a nodular, fully delimited fibrocartilaginous structure with regular echogenicity. This was also reported in a previous study by Burman and Lapidus^20^.

In the dissections, the IO showed a typical intractable keratosis plantar to the IPJ with or without associated (fascial) biomechanical alterations; these have also been described as cofactors for hyperpronation, functional hallux limitus, or hallux rigidus, associated with hallux extensus interphalangeus^21–23^. Despite conservative treatment, intractable plantar keratosis can be very disabling and require surgical intervention for fast recovery^21,23^. In view of its sensitivity and non-invasiveness, US could be recommended as a first option diagnostic tool rather than X-ray to confirm the diagnosis of an IO.

Since the 1970s, different approaches to IO exeresis have been described^24^. Few of them performed the so-called minimal incision (mini-open) procedure, published in 1982 and 1989^25,26^. Compared with the techniques reported in literature, our medial “ultra”-minimally invasive surgical approach, chosen because of its safety and the convenience of US guidance, protects all important anatomical structures^27^. During the last decade, some US-guided surgeries for other foot and ankle problems have been found to be safe and effective in both anatomical dissections of cadavers and clinical trials^28,29^. To the best of our knowledge, the present study describes the first ultra-minimally invasive US-guided technique with a medial approach for assessing an IO plasty. This technique, from our point of view, has a great advantage over open surgery. First, it is performed without ischemia, potentially reducing post-surgical pain and being particularly valuable for patients with higher morbidities (e.g., diabetics). Second, the incision is minimal (1.5 mm), classifying this surgical technique as “ultra-minimally invasive”. This causes minimal side effects in terms of fibrosis (which can entrap adjacent nerves), a better cosmetic result, less infection, less postoperative pain, and a faster recovery. Therefore, there is no need for a surgical cast or surgical shoe, and patients could be weight-bearing after the first day post-surgery. This US-guided technique is an excellent way to perform surgery with minimal injury to healthy tissue such as skin, fat, and fasciae. Our post-surgical recommendations are limited to wearing running shoes for the first week with a light dressing bandage, often associated with an orthotic device.

Compared to open surgery and MIS (fluoro-guided) surgeries, a US-guided approach has the advantage of direct control over the soft tissues throughout the procedure, which helped us to protect adjacent anatomical structures, especially the medial plantar nerve. Nerve injuries can lead to severe neuropathies as described by Mann and Wapner^21^. In a preclinical anatomical study, Le Corroller et al. also showed that the medial plantar proper digital nerve is visible adjacent to the first metatarsal phalangeal joint when US is used^30^. The authors of this study reported the possibility of visualizing similar small nerves, showing monofascicular features for the medial plantar nerve at foot and ankle revealed by US^31^. They also verified through dissections that the medial plantar nerve can be correctly visualized medial to the IPJ and that injuries to anatomical structures can be avoided during US-guided surgical procedures^28,31^.

A limitation of our study is the small sample size of 18 fresh frozen feet; further clinical studies, which are already in progress, are necessary to validate our results.

## Conclusion

Our results indicate that US is a better and more precise tool for diagnosing the IO of the IPJ than X-rays.

Moreover, our mini-invasive, US-guided technique was feasible and safe, at least in bodies donated to science.

## Data Availability

All data generated or analyzed during this study are included in this published article.
